# The Utility of Recycled Eyeglasses: A Pilot Study at the Los Angeles County Department of Health Services

**DOI:** 10.5195/ijms.2021.894

**Published:** 2021

**Authors:** Valerie P. Huang, Mary E. Kim, Sukriti Mohan, Lauren P. Daskivich, Jesse L. Berry

**Affiliations:** 1 Keck School of Medicine, University of Southern California, Los Angeles, CA, United States.; 2 Keck School of Medicine, University of Southern California, Los Angeles, CA, United States.; 3 Keck School of Medicine, University of Southern California, Los Angeles, CA, Unites States.; 4 Los Angeles County Department of Health Services, Los Angeles, CA, Unites States.; 5 Keck School of Medicine, University of Southern California & Children’s Hospital Los Angeles, Los Angeles, CA, United States.

**Keywords:** Refractive Error Development, Visual Acuity, Low Vision (Source: MeSH-NLM)

## Abstract

**Background:**

The cost of eyeglasses is variably covered by medical insurance and thus is a significant barrier for patients in lower socioeconomic classes. We evaluated the efficacy of Recycle Vision (RV) at LAC+USC Medical Center, a monthly clinic run by volunteer medical students that provides free donated eyeglasses.

**Methods:**

A convenience sample of 30 patients was surveyed from August 1, 2019 to December 31, 2019. Patients’ prescriptions were matched with available eyeglasses based on spherical equivalent and axis of astigmatism using Winglasses software algorithm; patients selected glasses from these options based on subjective improvement of vision. All participants consented to a phone follow-up survey 1 month after initial visit to gauge satisfaction with glasses and rate difficulty in completing daily activities pre- and post-RV visit on a scale of 1 to 5 (5 being the greatest), with a 100% response rate.

**Results:**

Of the 30 study participants, 90% received eyeglasses from RV, with reported improvement in ease of daily activities of 3.96. 67% of respondents stated that if RV clinic did not exist, they would not have obtained glasses elsewhere; cost was the most commonly (70%) cited barrier. Upon follow-up, average likelihood of patients referring friends/family to RV was 4.07 (SD 1.14).

**Conclusion:**

The majority of RV patients received free eyeglasses and had subsequent improvement in their quality of life. This pilot study demonstrates that programs offering free eyeglasses can effectively correct refractive error and can offer a practical public health solution to improve functionality for underserved populations.

## Background

Vision loss is the third most common medical impairment,^1^ with uncorrected refractive error being the leading cause of moderate or severe vision impairment.^2^ Uncorrected refractive error includes myopia (near-sightedness), hyperopia (far-sightedness), presbyopia (loss of near vision with age), and astigmatism (commonly from an irregularly shaped cornea). These types of vision impairment can be assessed through a simple eye examination and require little more than a pair of eyeglasses to correct. However, the cost of refractive eyeglasses is variably covered by insurance and can present a significant barrier for patients, especially those in lower socioeconomic classes.^3^ The World Health Organization estimates that 90% of the visually impaired live in low-income environments,^4^ and prior studies have illustrated that societal factors are consistently a barrier in correcting vision impairment.^5^ For example, Medi-Cal (California’s version of Medicaid) vision benefits include a routine eye examination every 24 months, but only patients under 21 years old and residents of nursing homes receive complete coverage of eyeglasses.^6^

One specific program created to eliminate the monetary barrier of obtaining glasses is the Recycle Vision clinic at the Los Angeles County + University of Southern California (LAC+USC) Medical Center Eye Clinic. Our patient population is primarily low-income and/or underinsured with limited access to care outside of the County health system. Recycle Vision is a monthly clinic run by volunteer medical students that provides donated eyeglasses for free.

The purpose of this pilot study was:

1. To evaluate the efficacy of Recycle Vision clinic services in reducing vision impairment
2. To quantify its effect on patients’ daily functioning
3. To determine patient satisfaction with receiving donated eyeglasses.

With these results, we hope to encourage other hospitals and clinics to implement similar programs for the visually impaired who do not have the financial means or access to obtain prescription eyeglasses.

## Methods

This is a patient quality of life survey study conducted on LAC+USC patients who received glasses from Recycle Vision clinics in the 4-month period from August 1, 2019 to December 30, 2019. These clinics are held once a month for patients of LAC+USC ophthalmology; all patients who visit Recycle Vision clinic with a current prescription seeking eyeglasses are seen. The Winglasses computer algorithm is used to suggest the closest approximate matches based on the patient’s spherical, cylindrical, and axis equivalent. Because the availability of glasses on-hand at Recycle Vision clinic is directly dependent on community donations, the number of potential matches can range from 3 to 10+ potential eyeglasses. Patients offered multiple choices of glasses based on optimization of the prescription parameters are then allowed to choose which pair of eyeglasses they feel best improves their vision impairment. This study met criteria outlined in the 45 CFR 46.104(d) category by the Department of Health and Human services meeting criteria for subjects’ research and was thus approved by the University of Southern California iSTAR Internal Review Board, and the methods were in accordance with the guidelines of Declaration of Helsinki. STROBE guidelines were followed as applicable to guarantee the quality of this observational study.^7^

### Data Collection and Analysis

Patients were asked if they were willing to participate in a short, written survey (Supplementary Material), and verbal consent was obtained. Patients were assured that this was a completely voluntary survey and that all information would be kept confidential and separate from their medical records; no demographics nor identifiable information was collected as part of the survey. All patients, regardless of survey participation, were trialed for a matching prescription eyeglasses through the services of Recycle Vision clinic.

The same day survey was conducted in English or Spanish, based on the preference of the patient. The consented patients were asked to list their phone number, so that they could be contacted for the one month follow up survey. Phone calls were completed by an author of this study (VH). The questions in the two surveys were either simple “yes/no” questions, or questions based on the Likert scale, a symmetric scale that is commonly used in survey-based studies. Survey questions can be seen in Table 1. Primary measured outcomes included quality of life as measured by patient-reported improvement in ease of daily activities with Recycle Vision eyeglasses, and patient-reported likelihood of recommending Recycle Vision services. Excel was utilized to calculate both descriptive and inferential statistical tests.

## Results

During the study period, 30 patients attended Recycle Vision clinic for eyeglasses services; all 30 patients consented and were included in this study. 100% of patients were successfully reached by phone for the second half of the survey, which was carried out between one to two months after the initial clinic visit. Of the 30 study participants, 90% (27/30) received a pair of glasses from Recycle Vision clinic and 10% (3/30) did not receive glasses due to lack of a suitable match.

**Figure 1.**
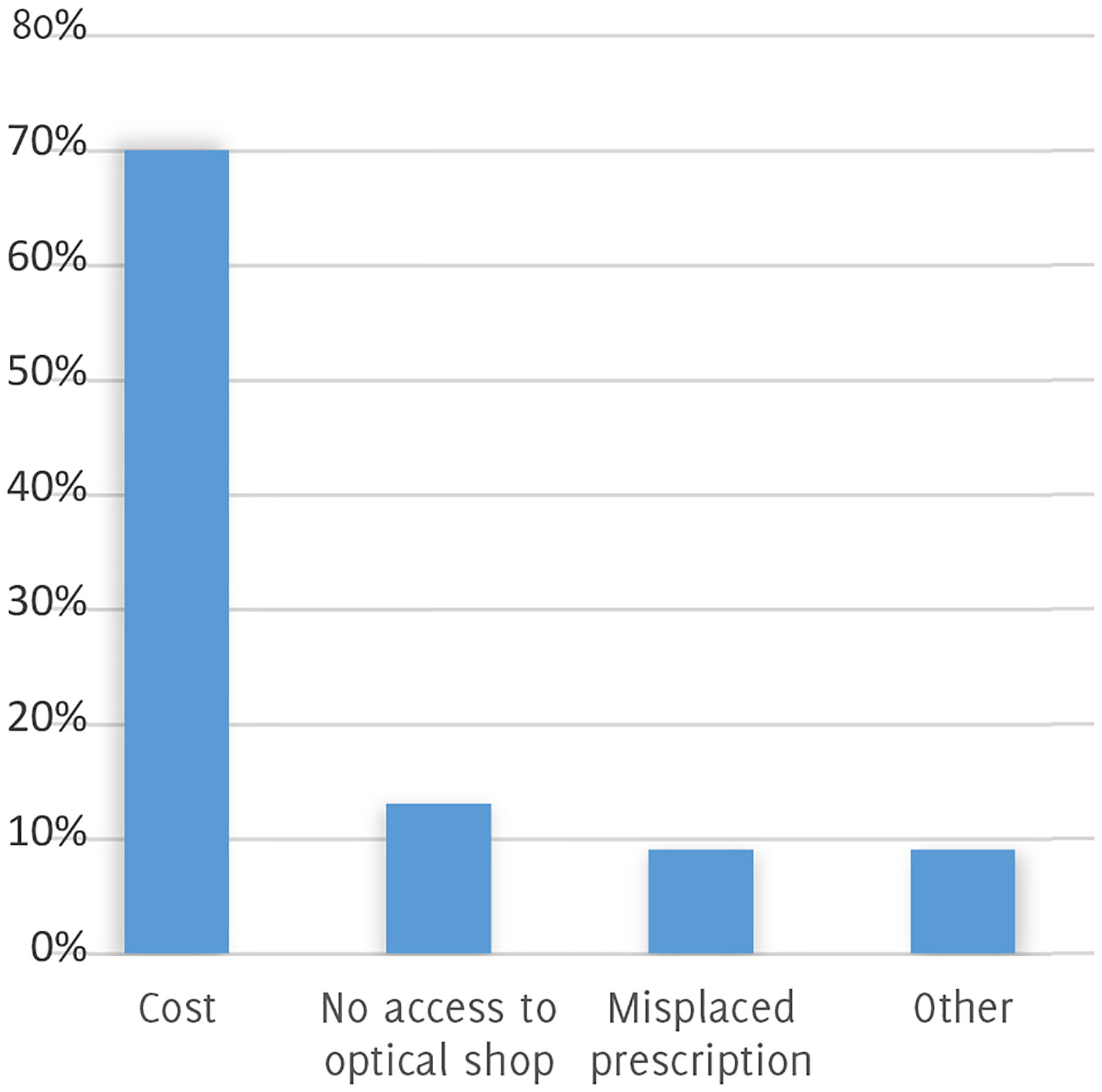
Reasons Cited by Patients for not Obtaining Glasses Elsewhere if Recycle Vision Clinic Was not an Option.

Of the surveyed patients, 43% (13/30) owned glasses prior to visiting Recycle Vision clinic. Clinic survey results, as well as descriptive statistics, are listed in Table 1. The mean level of self-reported improvement in ease of performing daily activities after receiving Recycle Vision glasses was 3.96 (on a scale of 1–5, with 5 being greatest), supported by participants reporting that they wore their glasses frequently and would be likely to recommend Recycle Vision clinic to others. Notably, 67% (20/30) patients responded that they would not have obtained glasses elsewhere outside of Recycle Vision clinic. Cost was the most common barrier, cited by 70% of survey respondents; other commonly cited reasons for this response are listed in Figure 1.

A Mann Whitney U test was performed to compare the mean difficulty in completing daily tasks between those who owned glasses prior to visiting RV clinic (n=13), and those who did not own glasses prior to visiting Recycle Vision Clinic (n=17); the resulting summed ranks for each patient group totaled to 235 and 431, respectively. The calculated test statistic indicates that there was no significant difference between the two groups (p=0.86). The observed standardized effect size was calculated to be 0.029.

A Mann Whitney U test was also performed to compare the mean improvement in completing daily activities as reported upon survey one month after visiting Recycle Vision clinic between those who owned glasses prior to visiting Recycle Vision clinic and those who did not; the resulting summed ranks for each patient group totaled to 183 and 224, respectively. The calculated test statistic indicates that there was no significant difference between the two groups (p=0.79). The observed standardized effect size was calculated to be 0.050.

## Discussion

Uncorrected refractive error is the most common cause of vision impairment worldwide, and the majority of those affected are of low socioeconomic status.^8^ LAC+USC Medical Center primarily serves these low-income patients, as evidenced by the fact that roughly 75% of our patient population utilizes Medi-Cal or is uninsured. Since January 2020, Medi-Cal vision benefits only cover the cost of eyeglasses for patients under 21 years old and residents of nursing homes.^6^ Unfortunately, there are only a few programs that offer eyeglasses at a discounted price in both developed and developing countries, such as the Scojo Foundation^9^ or the OneSight OnSite Voucher Program.^10^ These programs are still limited, as services that are redeemable online require an internet connection and a valid credit/debit card, both of which can be difficult to obtain for patients of underserved populations.

The results of our study show that over half (57%) of patients who attended Recycle Vision clinic during the study time period did not previously own glasses. Out of the 13 patients who previously owned glasses, 69% self-reported that their previous glasses did not suit their needs, supported by their average difficulty of 4.00 out of 5 in completing daily tasks. Across all participants, the mean level of self-reported improvement in ease of completing daily tasks was 3.96 out of 5 after receiving Recycle Vision glasses, suggesting that our clinic was able to improve their vision. Studies have shown that the resultant economic burden in daily decrease in productivity outweighs the cost of correcting refractive error.^11,12^ Thus, expansion of vision services such as Recycle Vision clinic for low-income patients could yield a net economic gain in daily household productivity and a reduction in unemployment numbers by patrons re-joining the workforce.^12^

The majority (53%) of surveyed patients indicated cost as the primary reason for not obtaining eyeglasses elsewhere. Previous studies have also found that lack of insurance or vision services coverage is directly related to the population’s unmet need for eyeglasses.^13^ However, since no insurance data was gathered to maintain anonymity, it is unclear if the limiting factor of cost of obtaining prescription eyeglasses is due specifically to lack of insurance coverage. For example, poor vision impairs one’s capacity to navigate and understand programs that provide low-cost vision care, but patients could misattribute this as services being inaccessible.^13^ Therefore, the lack of identifying demographic information prevents us from drawing conclusions about etiologies of identified barriers in obtaining prescription eyeglasses.

As this was a voluntary survey, one limitation of this study was inadvertently selecting for a biased group with positive responses not representative of the entire patient population. Additionally, we did not quantify each patients’ total degree of refractive error with and without glasses, so reported improvements in vision were not standardized. Regardless, patients indicated significant subjective improvement in their daily functioning along with comfort and frequent daily use of their Recycle Vision eyeglasses; this is supported by their high reported likelihood of recommending Recycle Vision services to others. Previous studies have demonstrated that self-reported data on eyeglass use and vision impairment are reliable,^14,15^ and this method aligned with our goal to evaluate patient satisfaction with recycled eyeglasses. Another limitation was that the Winglasses algorithm used in this study is proprietary and unable to be amended by the study authors; it takes into account prescription parameters from both eyes and attempts to find eyeglasses in the database that come close to an optimized value. Thus, eyeglass options that were offered to patients with severe uncorrected refractive error in only one eye were options that might subjectively worsen rather than improve vision overall. For procedure standardization, these patients were offered eyeglasses using the same algorithm. However, patients with drastically different prescriptions in each eye may benefit more from eyeglasses personalized to their exact prescription.

Lastly, this study was limited by small sample size, along with the fact that our surveyed population was all LAC+USC patients, which suggests a lower socioeconomic status than the general population. The effects of limited sample size were reflected in the results from the Mann Whitney U test. The calculated test statistic showed that there was no statistically significant difference in either the mean difficulty in completing tasks pre-clinic or in the mean improvement in completing daily tasks post-clinic between patients who previously owned glasses and patients who did not, suggesting that patients who owned glasses prior to Recycle Vision did not have up to date prescriptions and struggled equally as much as those who had no glasses at all. The results of Mann Whitney U test also showed that there was no significant difference in the mean improvement in completing daily activities between the participants who previously did and did not own glasses prior to visiting Recycle Vision clinic. It should be noted that LAC+USC is a tertiary care facility and as such, many patients who seek ophthalmologic care at these clinics have ocular disease in addition to simple refractive error. Because the survey used in this study did not incorporate questions that required patients to report the presence of presbyopia and the analysis did not quantitatively incorporate the improvement in visual acuity, our study cannot definitively report on whether prior ocular disease has an impact on the mean improvement in completing daily tasks. The low value of the calculated observed mean effect size illustrates the need for a larger sample size to reach statistical significance. However, we wanted to utilize preliminary results of this pilot study to illustrate the importance of these programs for underserved populations in seeking eyecare due to the relative paucity of current literature spotlighting these programs.

While these results may not be applicable to all eye clinics in the United States, they are useful in similar safety net patient populations and illustrate a problem with a simple solution. All patients in our study were referred to Recycle Vision clinic because they receive consistent eye care from LAC+USC but were unable to obtain glasses on their own. We hope that our patients’ reported satisfaction and improvement in daily functioning will encourage other institutions to implement similar programs. Thankfully, there are several other similar clinics that already exist.^16,17^ In future studies, we recommend larger sample sizes with longer follow-up to conclusively determine the long-term impact of clinics such as Recycle Vision. Additionally, we hope that future research can stratify patients, such as by the degree of refractive error, concurrent medical comorbidities, and socioeconomic and/or insurance status to better support programs that provide glasses for patients in lower socioeconomic classes with significant vision impairment.

## Supplementary Material

supp survey

## Figures and Tables

**Table 1.** Compiled Clinic Survey Results.

Characteristics	Mean (Standard Deviation) (scale of 1–5, 5 being greatest)	Number of Responses (n=30)
Number of participants who owned glasses pre-RV clinic		13 (43% of respondents)
Number of participants who did not own glasses pre-RV clinic		17 (57% of respondents)
Difficulty of completing daily tasks pre-RV for patients who previously owned glasses (scale of 1–5, 5 being most difficulty)	4.00 (SD 1.15)	13
Difficulty of completing daily tasks pre-RV for patients who did not own glasses (scale of 1–5, 5 being most difficult)	4.38 (SD 0.96)	17
Number of patients who stated that pre-RV glasses did not satisfy needs		9 (69% of respondents)
Comfort of new Recycle Vision (RV) glasses	3.59 (SD 1.23)	27
Reported frequency of wearing new RV glasses	3.81 (SD 1.21)	27
Amount of improvement in ease of daily tasks with new RV glasses	3.96 (SD 1.13)	27
Likelihood of recommending RV services	4.07 (SD 1.14)	30
