# Supplementary material for: The Utility of Recycled Eyeglasses: A Pilot Study at the Los Angeles County Department of Health Services
Source: Int J Med Stud. Author manuscript; Available in PMC 2022 Jul 1. (PMC9247889; doi:10.5195/ijms.2021.894)
Supplement: supp survey [file NIHMS1814233-supplement-supp_survey.pdf]

The Utility Of Recycled Eyeglasses: A Pilot Study At The Los Angeles County Department Of Health Services

Supplementary Material: Recycle Vision Patient Survey

Date (*fecha*): ____________

Phone number (*número de teléfono*): ___________________________

Recorded Prescription (*Prescripción grabada*): _____________

Prescription of Glasses obtained (*Prescripción de los anteojos recibidos*): ______________

* Please circle one answer option for each of the following questions. *
* *Por favor, circule una opción de respuesta para cada de las siguientes preguntas.* *

Did you own glasses before visiting the Recycle Vision Clinic? Y / N
¿Tenías anteojos antes de participar en la Clínica de Recycle Vision? Sí / No

If yes, please answer the following two questions:
Si ya tiene anteojos, por favor responde a las siguientes preguntas:
Do your previous glasses meet your needs? Y / N
¿Sus anteojos presentes los satisfice sus necesidades? Sí / No

On a scale of 1 to 5 (maximum), please rate how difficult it is to complete your daily tasks with your current pair of glasses on:
En una escala de 1 a 5, 5 es lo máximo, evalúe lo difícil que es completer sus tareas diarias cuando está vistiendo sus anteojos presentes:
5 – very difficult (*muy difícil*)
4 – mostly difficult (*un poco difícil*)
3 –neither difficult nor easy (*ni difícil ni fácil*)
2 – a little easy (*un poco fácil*)
1 – very easy (*muy fácil*)

If you did not own glasses before visiting the Recycle Vision Clinic, on a scale of 1 to 5 (maximum), please rate how difficult it is to complete your daily tasks (e.g. driving, cooking, reading) without glasses:
Si no tenia anteojos antes de participar en la Clínica de Recycle Vision, en una escala de 1 a 5, 5 es lo máximo, evalúe lo difícil que es completer sus tareas diarias (e.g. conducir, concinar, leer):
5 – very difficult (*muy difícil*)
4 – mostly difficult (*un poco difícil*)
3 –neither difficult nor easy (*ni difícil ni fácil*)
2 – a little easy (*un poco fácil*)
1 – very easy (*muy fácil*)

If we did not host a Recycle Vision Clinic to give out free glasses, would you have obtained eyeglasses elsewhere? Y / N
Si no temenos una Clinica de Recycle Vision, ¿habrías obtenido anteojos en otro lugar? Sí / No

If not, why not? Please circle at least one answer choice, at most two choices below.
Sí no, ¿por qué? Por favor, circule por lo mínimo una, por lo máximo does de las siguientes opciónes de respuesta.

A. Cost (*costo de anteojos*)
B. No access to an optical shop / do not know how to find a shop (*no tengo acceso a una tienda óptica / no sé como encontrar una tienda óptica*)
C. Do not like wearing eyeglasses (*no me gusta usar anteojos*)
D. Lost my prescription / do not know what it is (*no sé mi prescripción / no sé donde esta mi prescripción*)
E. Other (*otro razón*)

Please answer the following questions 1 month after receiving glasses from Recycle Vision Clinic, on: (date)
Por favor, responda a las siguientes preguntas un mes después de recibir los anteojos de la Clinica Recycle Vision en: (date)
On a scale of 1 to 5 (most comfortable), please rate how comfortable your glasses are:
En una escala de 1 a 5, 5 es lo máximo, por favor evalúe la comodidad de los anteojos:
5 – very comfortable (*muy cómodo*)
4 – mostly comfortable (*un poco cómodo*)
3 – I am neither comfortable nor uncomfortable (*no estoy ni cómodo ni incómodo*)
2 – a little uncomfortable (*un poco incómodo*)
1 – very uncomfortable (*muy incómodo*)

On a scale of 1 to 5 (maximum), please rate how often you wear your glasses:
En una escala de 1 a 5, 5 es lo máximo, evalúe la frecuencia con que usa sus anteojos:
5 – all the time (*siempre*)
4 – most of the time (*la mayoría del tiempo*)
3 – sometimes (*a veces*)
2 – rarely (*raramente*)
1 – never (*nunca*)

On a scale of 1 to 5 (maximum), please rate the amount of improvement in your daily functioning since obtaining free glasses from Recycle Vision:
En una escala de 1 a 5, 5 es lo máximo, evalúe si hubo una mejora significada en su funcionamiento diario desde la obtenación de anteojos gratis de Recycle Vision:
5 – a lot of improvement (*mucha mejora*)
4 – some improvement (*un poco mejora*)
3 – no change (*es el mismo*)
2 – somewhat worse (*un poco peor*)
1 – much worse (*mucho peor*)

How likely are you to recommend the services of Recycle Vision to a family member?
¿Qué tan probable es que recomiende los servicios de Recycle Vision a un miembro de la familia?
5 – very likely (*muy probable*)
4 – somewhat likely (*probable*)
3 – neither likely nor unlikely (*ni probable ni improbable*)
2 – unlikely (*improbable*)
1 – very unlikely (*muy improbable*)

Cite as
Valerie P. Huang VP, Kim ME, Mohan S, Daskivich LP, Berry JL. The Utility Of Recycled Eyeglasses: A Pilot Study At The Los Angeles County Department Of Health Services. Supplementary Material: Recycle Vision Patient Survey. Int J Med Students. 2021 Jan-Apr;9(1):41-2.

This work is licensed under a Creative Commons Attribution 4.0 International License

ISSN 2076-6327
This journal is published by the University Library System, University of Pittsburgh as part of the Digital Publishing Program and is co-sponsored by the University of Pittsburgh Press.
